# Dementia Diagnosis and Challenges in Minority Populations

**DOI:** 10.1093/geroni/igaa057.525

**Published:** 2020-12-16

**Authors:** Halima Amjad, Marcela Blinka, Jennifer Aufill, Quincy Samus

**Affiliations:** 1 Johns Hopkins University, Baltimore, Maryland, United States; 2 Johns Hopkins University, School of Medicine, Baltimore, Maryland, United States; 3 Johns Hopkins Bloomberg School of Public Health, Baltimore, Maryland, United States

## Abstract

Alzheimer’s disease and related dementias are underdiagnosed in the United States, with potentially higher rates of underdiagnosis among minority groups. Our objective was to examine perceptions of dementia, the utility and timeliness of diagnosis, and experiences obtaining diagnosis and care in minorities. We recruited 17 family caregivers of African American (n=11), Latino (n=3), and Asian (n=3) persons with dementia (PWD) to complete surveys and semi-structured interviews. Caregivers were mostly female (n=14), children of PWD (n=14), and had greater than high school education (n=16). Mean PWD age at diagnosis was 76 years (range 63-90) with mean 17 months from symptom observation to diagnosis (range 0.5-36 months). Interview themes were coded using a grounded theory approach. Emerging themes related to concerns prior to diagnosis, diagnosis experiences, timeliness of diagnosis, ways to improve diagnosis and care, familiarity with dementia, and stigma. Poor memory was the most common early concern; caregivers also noted behavioral symptoms, weight loss, family stress, and PWD vulnerability. Caregivers recalled key moments when they knew something was wrong. Primary care was the most frequent starting point in diagnosis; longstanding primary care relationships both facilitated and hindered diagnosis. Nine of the 17 caregivers felt diagnosis was delayed. Caregivers preferred clinicians who were forthcoming with the diagnosis and what to expect and noted the importance of family meetings or counseling. Prior experience or knowledge of dementia was common. Caregiver perspectives and experiences elicited in this study may be translated to interventions and clinical practices that proactively detect and address dementia in minorities.

